# Climate Change and Green Sea Turtle Sex Ratio—Preventing Possible Extinction

**DOI:** 10.3390/genes11050588

**Published:** 2020-05-25

**Authors:** Jana Blechschmidt, Meike J. Wittmann, Chantal Blüml

**Affiliations:** Faculty of Biology, Theoretical Biology, Bielefeld University, Universitätsstraße 25, 33615 Bielefeld, Germany; jana.blechschmidt@uni-bielefeld.de (J.B.); meike.wittmann@uni-bielefeld.de (M.J.W.)

**Keywords:** evolutionary rescue, global warming, rapid evolution, *Chelonia mydas*, temperature-dependent sex determination

## Abstract

Climate change poses a threat to species with temperature-dependent sex determination (TSD). A recent study on green sea turtles (*Chelonia mydas)* at the northern Great Barrier Reef (GBR) showed a highly female-skewed sex ratio with almost all juvenile turtles being female. This shortage of males might eventually cause population extinction, unless rapid evolutionary rescue, migration, range shifts, or conservation efforts ensure a sufficient number of males. We built a stochastic individual-based model inspired by *C. mydas* but potentially transferrable to other species with TSD. Pivotal temperature, nest depth, and shading were evolvable traits. Additionally, we considered the effect of crossbreeding between northern and southern GBR, nest site philopatry, and conservation efforts. Among the evolvable traits, nest depth was the most likely to rescue the population, but even here the warmer climate change scenarios led to extinction. We expected turtles to choose colder beaches under rising temperatures, but surprisingly, nest site philopatry did not improve persistence. Conservation efforts promoted population survival and did not preclude trait evolution. Although extra information is needed to make reliable predictions for the fate of green sea turtles, our results illustrate how evolution can shape the fate of long lived, vulnerable species in the face of climate change.

## 1. Introduction

Global warming poses a potential threat to biodiversity all over the world. A group of species at particular risk are long-lived reptile species with temperature-dependent sex determination (TSD) [1,2]. In these species, increases in temperature can lead to biased sex ratios. Shortage of one sex is then expected to lead to mate-finding difficulties, failure to reproduce, and ultimately population decline [3]. This can be seen as a mate-finding Allee effect [4]. The persistence of these species will depend on whether or not they are able to adjust rapidly enough to the increasing temperatures to prevent extinction. There are multiple traits and behaviours that might evolve to counteract negative effects of climate change. For example, some species may shift their geographical range or phenology, although this may be restricted by the availability of suitable habitat and by seasonality in other environmental factors. Here, we consider rapid evolution of traits influencing the sex determination system as a potential route of evolutionary rescue in the face of climate change [5].

The green sea turtle (*Chelonia mydas*) is an example of a long-lived species where survival of populations may depend on rapid adaptation to increasing temperatures [6]. Females express nest site philopatry, that is, they come back to the same beach where they were born and deposit four to five nests with about 100 eggs per nest, which are covered with sand [7,8]. The embryo’s sex is determined during the second third of incubation [9,10,11]. The warmer the egg, the higher the probability of a female hatchling. Furthermore, very high temperatures increase egg mortality [12]. The pivotal temperature is defined as the temperature at which 50% of the hatchlings are female. Pivotal temperatures vary across species and between populations [9,13,14]. For *C. mydas*, pivotal temperature could range between 20.0 °C and 30.3 °C on the basis of field measures in Costa Rica [15,16]. For Heron Island in the southern Great Barrier Reef (sGBR), the pivotal temperature is 27.6 °C [17]. Here, we focus on the northern Great Barrier Reef (nGBR) population, which has a pivotal temperature of 29.3 °C [17]. A slight change in temperature can alter the sex ratio of turtle hatchlings substantially. Only 1 °C above the pivotal temperature, 80% of hatchlings are female [17]. Global mean temperatures, however, are expected to increase by 1.0 °C to 3.7 °C until 2100 [18]. Within the lifespan of an individual turtle, the temperature might have already increased by 0.8 °C [18]. The oldest turtle fossil records date back to the Triassic (120 million years), indicating that turtles have survived several climate fluctuations in the past [19,20]. However, it is unclear whether the species will be able to keep up with the high pace of current climate change.

The nGBR population is one of largest breeding green sea turtle populations in the world. It has over 200,000 breeding females [21]. Currently this population has an approximate overall sex ratio of 80% female, with 99% of the non-adult turtles being female [21]. It has been suggested that a female-biased sex-ratio may enhance population growth [22], as few males can fertilize many females. Moreover, although females can only mate about every three years, and store sperm throughout a season, males are able to mate every year [8,23,24]. Thus, the operational sex ratio will not be as skewed as the overall sex ratio in the population, and thus a higher female skewed sex ratio is not necessarily bad per se [25]. However, the low generation turnover rate may lead to a delayed impact of the lack of males. Eventually, the few remaining males may no longer be able to fertilize enough females to sustain the population, and the population might go extinct [26], especially if temperatures keep rising under climate change. Unfortunately, there is currently not much information on how female fertilization probability depends on sex ratio or the number of males in the population. 

A recent study suggests that juvenile recruitment in the nGBR population has decreased in the last few years [27]. Adaptation to higher temperatures could be necessary for this population to avoid extinction. Because evolutionary rescue is more likely in large populations [28], the nGBR population may be a good candidate for evolutionary rescue. With increasing bias in the sex ratio, a trait controlling offspring sex ratio would be under strong selection pressure. Depending on the frequency of males and females within the population, selection would favour the less common sex according to frequency-dependent selection [29]. For example, in a highly female-biased population, a trait leading to a production of more males would have an advantage. In green sea turtles, selection could act on a number of traits that influence egg incubation temperature or pivotal temperature and thus influence hatchling sex ratio. We here consider four such traits that have some empirical support for green sea turtles:

1. Nest depth. Green sea turtle females bury their eggs anywhere from 30 to 90 cm deep into the sand [7]. When comparing nests at different depths, deeper nests are on average cooler than more shallow nests [30]. The exact temperature depends on many factors, such as beach vegetation; wind; and sand grain characteristics such as size, colour, and moisture [31,32,33,34]. Moreover, shallow nests experience stronger temperature fluctuations than deeper nests [30].
2. Shading of nest. On a typical nesting beach, there is substantial vegetation that provides shade throughout the day [7]. Depending on the hours of direct sunlight that the nest receives, temperatures vary [35]. The coolest nests are those directly underneath a tree or bush because they will be exposed to the sun for the shortest amount of time. Too much vegetation, however, can also be detrimental because roots may deter turtles from digging their nests [35]. The mean nest temperature at a medium level of shade (15%) is 1 °C cooler than for nests located in the sun, and nests with a high level of shade (30%) were found to be 1.9 °C cooler [35]. These temperature differences could potentially have an effect on hatchling sex ratio. It has been proposed that a nesting site with high levels of vegetation and thus shaded areas has a high resistance to warming temperatures caused by climate change [36].
3. Pivotal temperature. The pivotal temperature varies between species with TSD and between populations of the same species [13]. Heritable variation in the sex-determination threshold has also been found within populations in other turtle species [37]. The genetic basis of TSD is still a topic of research, and probably involves multiple loci. The best-studied locus is the cold-inducible RNA-binding protein (CIRBP) gene whose expression differs in embryonic gonads at different temperatures [38], but additional loci may affect pivotal temperatures, for example those involved in the regulation of the aromatase gene [39].
4. Choice of nesting beach. Green sea turtles display maternal nest-site philopatry [7]. When a female reaches sexual maturity, she returns to her natal beach for oviposition. Different beaches can have different temperature conditions, depending on sand colour, grain size, and vegetation [34,40]. Additionally, the orientation of the beach on the island may also be important [35].

There are of course additional physiological and behavioural traits that could evolve in response to changing temperatures. One example is a shift in breeding season. Some studies have found that other turtle species are somewhat plastic in their nesting behaviour, and will start breeding at an earlier date when winters have been warm [41,42]. However, there is no evidence that *C. mydas* shift their breeding seasons in response to winter temperatures [43]. It has been suggested that many populations of loggerhead turtles, *Caretta caretta*, will not be able to shift their breeding seasons at a pace high enough to keep up with climate change [44]. In addition to all these limitations, it is not clear how an earlier onset of breeding season affects temperatures at different nest depths. Although in spring, shallower nests may be warmer, the opposite might be true for autumn, when the deep sand has been heated up by summer and shallow nests are becoming cooler [45].

Because climate change is occurring at a very fast rate compared to historical temperature changes, evolutionary adaptations would have to occur rapidly to rescue green sea turtles at the nGBR [18]. Because of slow generation turnover, it has been doubted as to whether evolution can be fast enough [21]. Should the turtles not be able to adapt to the rising temperatures, artificially lowering nest temperatures may hypothetically help to ensure population survival. Possible measures include protecting beaches that produce a higher ratio of males, creating artificial beaches or altering existing beaches by adding different sediment, such as lighter sand of different grain size that does not heat up as much, or moving nests to other beaches with cooler temperatures after oviposition [46]. We chose here to model the effect of manipulating nest depth as well as altering the level of shade of a nest. This then allows us to directly compare the effect of letting these traits evolve naturally and to anthropogenically manipulate them. Relocating nests deeper into the sand or providing them with shade should lead to an increase in male hatchlings. However, it is important that anthropogenic conservation efforts do not prevent evolution of the two traits in the long term. 

The population at the southern Great Barrier Reef (sGBR) appears to be less susceptible to climate change than the northern population [21]. It is located further away from the equator and hence does not experience equally high temperatures as the nGBR population. As a consequence, the sex ratio of the southern population is not as skewed as the northern one—it currently is 67% female [21]. Thus, dispersal of males from the sGBR may additionally promote the persistence of the nGBR population. Males from the sGBR population could keep the nGBR population from experiencing a lack of males, even if the population consists exclusively of females [21]. It is known that some level of migration takes place between the two populations [24]. However, the extent of crossbreeding remains unknown.

We created a stochastic individual-based model that includes all the above-mentioned evolutionary traits as well as anthropogenic conservation efforts. Our goal was to analyse how nest depth, shading, beach philopatry, and pivotal temperature influence sex ratios and population size in the face of climate change. Additionally, we explored how conservation measures could be used to maintain the nGBR population. Lastly, we quantified the effect of crossbreeding between the nGBR and sGBR populations.

## 2. Material and Methods

We built an individual-based stochastic model with overlapping generations. The model’s main purpose was to predict the survival probability and the sex ratio of the nGBR green sea turtle population under different climate, evolutionary, and conservation scenarios. In the first part of the study, we considered the fate of the population under constant temperatures between 25 °C and 38 °C. In the second part, we modelled four different temperature trajectories over the next centuries, on the basis of the predictions of the Fifth Assessment Report of the Intergovernmental Panel on Climate Change (IPCC) [18,47]. The model comprises the timespan between 1800 and 2500. 

The climate change model started in 1800 to give the population time to equilibrate. As we were unable to find any literature regarding historical sex ratios of green sea turtles, for the first 82 years, the baseline nest temperature was kept constant at 29.3 °C, which was equal to the pivotal temperature. This was based on the assumption that pre-industrial temperatures on average produced a 50:50 sex ratio, as would be expected on the basis of the work of Fisher [29]. This assumption should only have a minor effect on the results, however, as it mainly affects the starting population. Starting in 1883 (year 83 in the simulation) when temperature data became available, regional weather data from the International Comprehensive Ocean-Atmosphere Data Set ICOADS dataset was used [48]. Taking Jensen et al. [21] as oriented, we used mean monthly air temperatures available on a 1° scale with the coordinates 7.5°S to 12.5°S and 142.5°E to 144.5°E for 1960 to 2019 and a 2° scale with the coordinates 7°S to 13°S and 142°E to 145°E from 1883 to 1959 [21]. Temperatures were averaged over December (of the respective preceding year) to March, the main breeding season of *C. mydas* in the GBR [21]. The average nest temperature is some degrees warmer, mainly due to metabolic heating of the nest [10,41,49]. We calculated expected nest temperatures by adding 1.325 °C to the air temperature on the basis of the degree of metabolic heating in *C. mydas* nests reviewed in Howard et al. [10], as it appears that sand temperature at nest depth is very close to air temperature for this region [21,34,42].

From 2020 onwards, long-term temperature anomaly predictions relative to 1986–2005 were used to determine the average temperature [18,47]. We used the four different IPCC representative concentration pathways (RCP) scenarios RCP2.6, RCP4.5, RCP6, and RCP8.5. These were based on four different radiative forcing scenarios (the number representing W/m^2^ by 2100), each depending on the level of CO_2_ emissions (in the following referred to as constant, low, moderate, and high emission scenarios).

**Figure 1:**
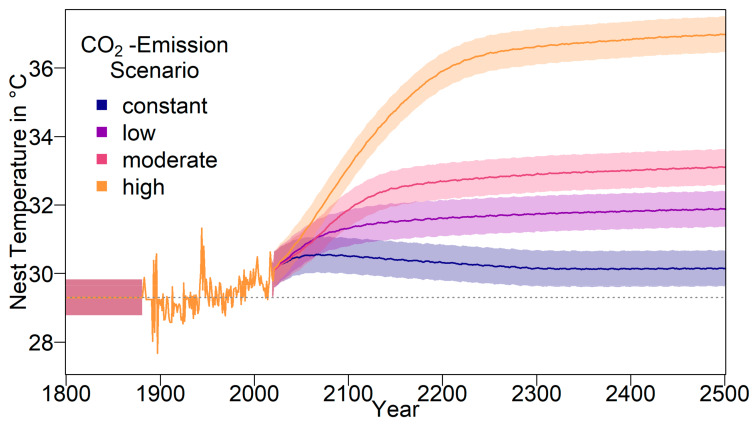
Nest temperature used for the simulation. Before weather data became available (1800-1882), the average nest temperature was assumed to be at 29.3 °C (pivotal temperature for northern Great Barrier Reef (nGBR) green sea turtle population). From 1883 to 2019, weather data were used [48]. Values are derived from the average air temperature during December to March in the corresponding region. Predicted temperatures starting in 2020 are derived from the Intergovernmental Panel on Climate Change (IPCC) report [18,47]. Shaded areas indicate the standard deviation of weather data, which was used for generating interannual variability by drawing simulated temperatures from a normal distribution during the implied intervals.

The resulting average baseline nest temperature trajectories can be seen in Figure 1. In order to obtain realistic year-to-year temperature variability, we drew temperatures for all years without available weather data (before 1883 and from 2020 onwards) from a normal distribution. The means were the temperatures described above for the respective years. For the standard deviation, we used the standard deviation of recorded temperatures from 1883 to 2019, which was 0.5234968. The standard deviation is indicated by shaded areas in Figure 1. 

For each climate change scenario, we let the genes for nest depth, level of shade, pivotal temperature, or beach orientation evolve, as well as all combinations of those parameters with and without migration and conservation. We ran 100 replicates per setup. 

On the basis of Limpus [17], sex ratio of hatchlings as a function of nest temperature, *t*, is described by Equation (1):

(1) $$f\left(t\right)=\frac{a}{1+e^{\frac{-\left(t-t_{piv}\right)}{b}}},$$

where *t_piv_* = 29.3 is the pivotal temperature, *a* = 1, and *b* = 0.4424779. Here, we used the information that *f*(29.3) = 0.5 and *f*(28) = 0.05 and solved the resulting system of two equations for the two unknowns *a* and *b* (see Appendix A for details). With these parameter values, the transitional range of temperatures (TRT), that is, the difference between the temperatures where 95% of hatchlings were female and the temperature where 5% of individuals were female, was 2.6 °C.

**Figure A1:**
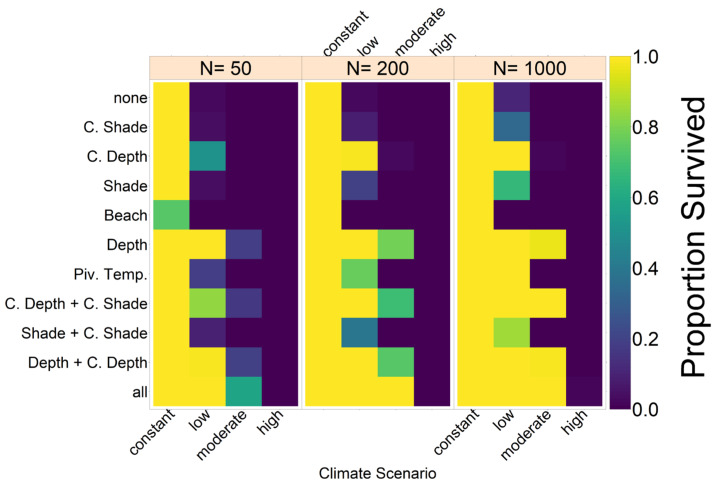
Effect of carrying capacity and thereby initial population size. Colours indicate the proportion of populations surviving to the year 2500. Note that the number of mating areas *g* was always half the carrying capacity to carrying capacity for this simulation. Varying the carrying capacity did not have a noticeable effect on population survival in the various setups.

Adult population size was variable but bounded by a carrying capacity *K* = 200. We assumed an initial population size *N_0_* = *K*. Note that for the sake of computational efficiently, our simulated populations were much smaller than the actual populations in nature. Supplementary simulations (please see Supplementary Materials) confirmed that our results are robust to changes in carrying capacity and initial population size (see Figure A1). 

**Figure A2:**
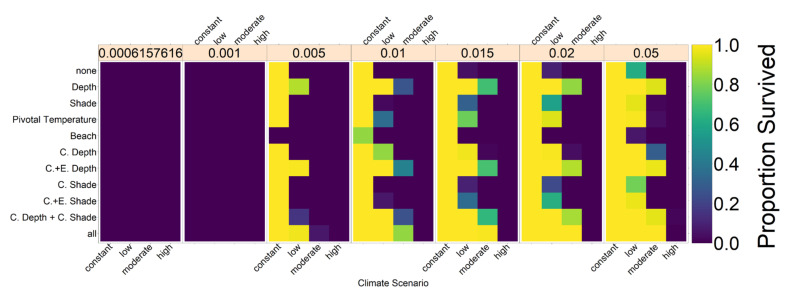
Robustness analysis for juvenile mortality, that is, the survival probability from egg to mature adult. Colours indicate the proportion of replicates in which the population survived up until year 2500. The leftmost box shows survival rate based on Chaloupka [50], where juvenile survival probability was chosen to obtain stable populations, but with a different set of parameters and assumptions.

A female in our model produced 100 hatchlings per breeding season if she found a mate and did not reproduce in the two prior years. Note that this was a rough estimate based on the fact that females lay multiple clutches of around 100 eggs per year but only a (largely unknown) fraction of eggs produces hatchlings [17]. Individual survival in the model was affected by several processes. Only 1.5% of hatchlings survive to the age of 40 [23], at which point they reach sexual maturity [21] and were included in our population size count. What mattered for our model was the number of offspring surviving to maturity. Because there was a lot of uncertainty in the literature regarding this number, we included the proportion of surviving offspring in a robustness analysis (Figure A2). Limpus suggests density dependence of hatching success for this species [17]. Thus, the number of offspring was additionally reduced according to carrying capacity (Equation (2)) by

(2) $$p_{survival}=min\left(\frac{K-N}{E},1\right),$$

where *E* is the total number of juveniles that would reach maturity in a given year. Adult turtles have a yearly survival probability *p_adult_* = 0.9482 as estimated by Chaloupka [50]. In nature, egg survival furthermore decreases at extreme temperatures [12], which was however not included in the model.

Each female has a chance of meeting a male each year as long as she has not reproduced in the two preceding years [8,23,24]. There is currently little information on how males and females encounter each other, but it is commonly assumed that these turtles mate in designated mating areas [51]. Here, we assumed that the probability for a female to encounter a male in a given year increased with the number of males in the population, *N_males_*, according to the Equation (3):

(3) $$p_{encounter}=1-{\left(1-\frac{1}{g}\right)}^{N_{males}},$$

**Figure A3:**
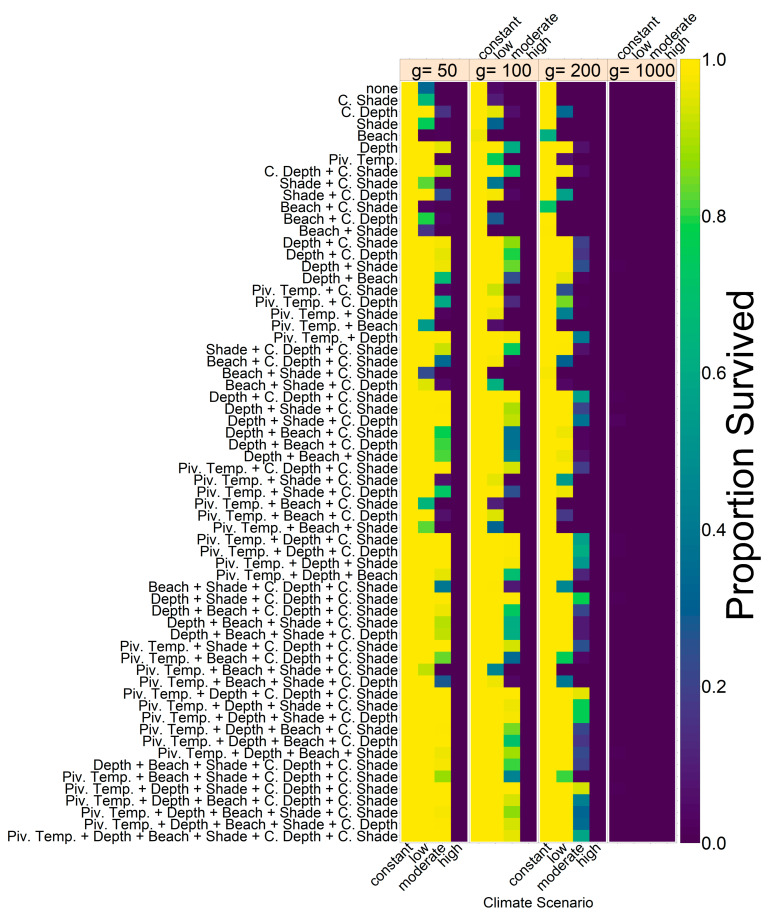
Effect of number of mating areas, *g*. The default value used for all other results is *g = 100*. This figure shows a summary for all possible combinations of trait evolution, philopatry, and conservation measures. Colours indicate the proportion of populations surviving to the year 2500. The more mating areas there were, the less likely the population was to survive, as the probability of finding a mate decreased with the increasing number of mating areas.

That is, the probability to not encounter a male decreased exponentially with increasing number of available males, with the rate of decrease determined by *g*. The parameter *g* can be interpreted as a number of mating areas if a male and female must randomly choose the same mating area in order to mate. *p_encounter_* can then be understood as 1 minus the probability that all *N_males_* males in the population go to a different mating area than the one chosen by the focal female. We chose *g* = 100 as the default value, which would give an encounter probability of 0.63 at a population size of 200 with a 50:50 sex ratio. In Figure A3, we explore the sensitivity of our results to the number of mating areas.

We made the assumption that there is a genetic basis to preferred nest depth, level of shade, and pivotal temperature. The genetics of the population were modelled as follows. Each individual had three diploid loci with two alleles each. There was one genetic locus each for nest depth, level of shade of the nest, and pivotal temperature, but only those relevant for the respective setup were taken into account to determine nest temperature and sex ratio. These traits were represented by real positive values between 0 and 1. Allele values for individuals in the initial population were independently drawn from a uniform distribution between 0 and 1. The mean of both of an individual’s alleles for a given trait was the phenotypically expressed trait for that individual. For example, a phenotype of 0.3 for the nest depth trait would mean that the female will bury her eggs at a depth of 30 cm in our model, and a trait of 0.8 would correspond to a nest depth of 80 cm.

Furthermore, each individual had a nesting beach, which depended on where it hatched, and therefore was a maternal effect.

The initial sex ratio of the starting population was 0.5 and individuals were evenly distributed over hatching beaches. Juveniles maturing within the first 40 years were generated in the same way as the rest of the starting population. This meant each of these initial cohorts of juveniles had a 50:50 sex ratio and a third of them hatched on each beach. Ages of juveniles were uniformly distributed between 1 and 39. We generated K*5 juveniles per year for the model to draw offspring from within the first 39 years. From this pool, maturing animals were drawn during the first 39 years of the simulation. Each individual had a chance to be added to the adult population as described by Equation (2). By running the simulation for a number of years prior to weather recordings and climate change, we ensured that the initial conditions did not influence the population’s response to climate change.

The genetic traits are inherited from both parents. For each trait, one of the mother’s alleles and one of the father’s alleles is randomly chosen for the offspring. When being passed on, alleles are susceptible to mutation. Mutation was implemented by drawing the new allele value from a Beta distribution. The two shape parameters, α and β, were calculated from the variance and mean of the distribution, as shown in Equations (4) and (5). The mean of the distribution, *µ*, was set to the original allele value. To our knowledge there are no empirical data on mutation processes in *C. mydas*, so we chose a standard deviation of 0.01, which led to the variance *σ*^2^ = 0.0001. α and β then follow from

(4) $$\alpha =\left(\frac{1-\mu }{\sigma^{2}}-\frac{1}{\mu }\right)\mu ²$$

and

(5) $$\beta =\alpha \left(\frac{1}{\mu }-1\right).$$

**Figure A4:**
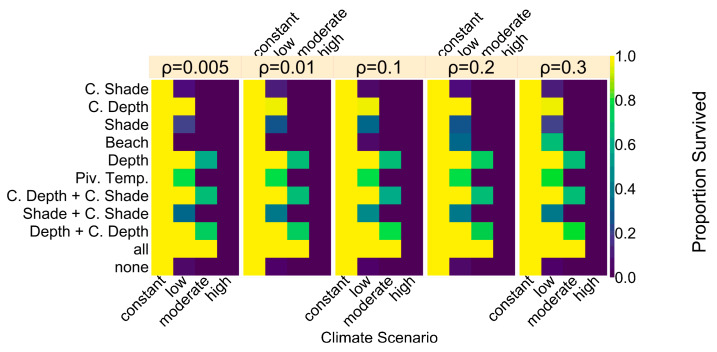
Effect of level of nest site philopatry 1-ρ. Numbers in orange boxes indicate the ratio of females that did not return to their native beach. Colours indicate the proportion of populations surviving to the year 2500.

If the drawn trait value was below 0.001 or above 0.999, the value was set to these boundary values, because for values of 1 or 0, the β distribution would break down. For the beach orientation, each individual had a chance of ρ = 0.005 of changing their beach orientation trait value to one of the other two possible beach orientations. Results for an altered ρ can be found in Figure A4.

Depending on the mother’s traits, the nest temperature will be warmer or colder than the temperature given by the environment. All effects of traits were added up and then added to the current year’s baseline nest temperature. 

Results from measuring nest temperatures at differing depths showed a linear relationship with a decrease of 5.6 °C per m on the basis of the work of Booth and Astill (2001) [52]. We used this to obtain Equation (6), and determine the temperature difference ∆*t* compared to the environmental temperature:

(6) $$\Delta t=5.6\cdot \left(0.5-depth\left[m\right]\right).$$

In our model, the depth of the nest was assumed to be determined by an individual’s genetics as described above, allowing a maximum temperature difference of 5.6 °C between the deepest and the shallowest nest. The default depth assumed in scenarios without depth evolution was 0.5 m. When nest depth was evolvable, eggs at 0.5 m (genetic trait value 0.5) experienced the corresponding year’s nest temperature without adjustments, whereas they could incubate at temperatures 2.8 °C warmer or colder depending on the mother’s trait value (when trait value was 1, then ∆*t* = −2.8 °C; when trait value was 0, ∆*t* = +2.8 °C).

Secondly, a nest’s level of shade influences its temperature [35]. The temperature difference compared to the baseline temperature for the model (Equation (7)) can be described as

(7) $$\Delta t=0.06\cdot \left(15-shade\left[\%\right]\right).$$

An individual’s preferred degree of shade for its nest will be determined by the two alleles in the same way as for the nest depth. Here, the genetic trait value, which was between 0 and 1, was multiplied by 30 to get the percentage of shade coverage. With 15% shade, nest temperature was equal to the year’s baseline temperature, 0% shade increased nest temperature by 0.9°C, and 30% shade reduced nest temperature by 0.9°C. Shading was limited to vary between 0–30% in the model, as that is the range covered by empirical data [35]. It might be possible to have higher levels of shade in nature, but the resulting nest temperature remains to be empirically investigated.

**Figure 2:**
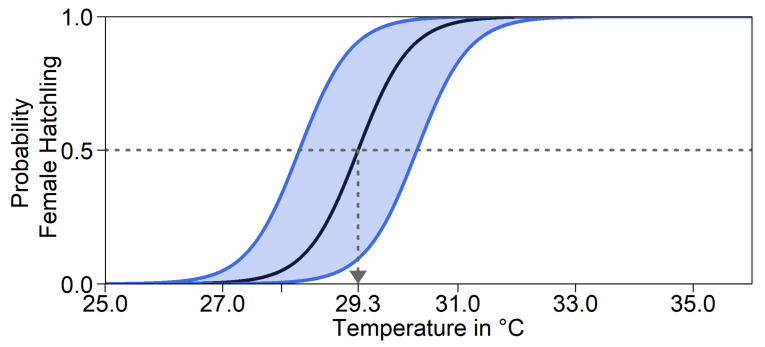
Probability of a female hatchling depending on temperature. The black line shows the result of Equation (1) [17]. The pivotal temperature 29.3 °C is indicated by the grey arrow. The blue lines are shifted by 1°C to the left or right, the maximum possible shift in our model. When the curve is shifted to the right, the pivotal temperature is higher, that is, there are more male hatchlings at higher temperatures. When shifted to the left, pivotal temperature is reduced, and more females hatch at colder temperatures. The shaded area indicates the range used in the model.

Furthermore, there might be a shift in pivotal temperature. This mechanism does not influence the nest temperature itself. Note that although the mother’s genes determine the depth and level of shade at which she buries the eggs, enzymes controlling offspring sex are produced within the egg. We therefore took the offspring’s genes into account to calculate its probability of developing into either sex. As illustrated in Figure 2, the same nest temperature will lead to different hatchling sex ratios, depending on the alleles. For a trait value of 0.5, the pivotal temperature was 29.3°C. In the model, the pivotal temperature could be shifted by at most 1°C in either direction for a trait value of 0 and 1. This was the average recorded difference in pivotal temperature between two populations of *Caretta caretta* in Australia [53]. We were unable to find any empirical data on variation in pivotal temperature within a population in *C. mydas*. For simulation runs without evolution of pivotal temperature, all individuals acted as if they had a trait value of 0.5. Throughout, we assumed that the parameters *a* and *b* of Equation (1), and thus the transitional range of temperatures, were constant.

Lastly, nest temperature is also influenced by beach orientation. Beach temperatures differed and were based on the work of Booth and Freeman, who measured nest temperatures on three beaches on Heron Island, which is located in the sGBR region [45]. In our model, we assumed that temperature of nests on the eastern beach represented the average baseline nest temperature for that year. We assigned each individual one of the nesting beach orientations on the basis of their beach of birth. Females later returned there, and the corresponding temperature was then applied to their nests, altering the temperature by +0.7 °C (north), 0 °C (east), or −0.9 °C (south) [45].

**Figure A5:**
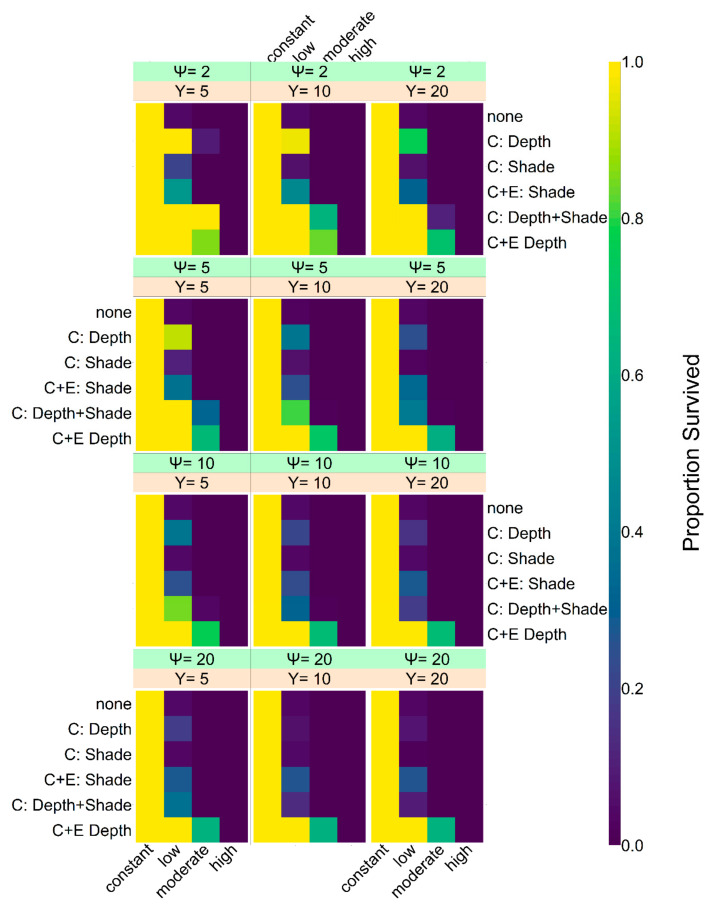
Effect of conservation measures. Υ is the interval at which efforts were carried out, for example, Υ = 10 means that the efforts were carried out every 10 years. Ψ is the frequency at which nests are manipulated, for example, Ψ = 2 means every second nest was manipulated. Colours indicate the proportion of populations surviving to the year 2500.

As level of shade and nest depth seem easiest to manipulate in the field, we also included a setup where we tested the influence of conservation efforts on them. In the corresponding simulations, nest depth was changed to 0.9 m, independently of genetics, and shade level was increased to 30%. We decided to manipulate every second nest (*Ψ* = 2) every 10 years (*Υ* = 10) as default condition, starting in 2020. These parameters were also altered, and results can be found in Figure A5.

**Figure A6:**
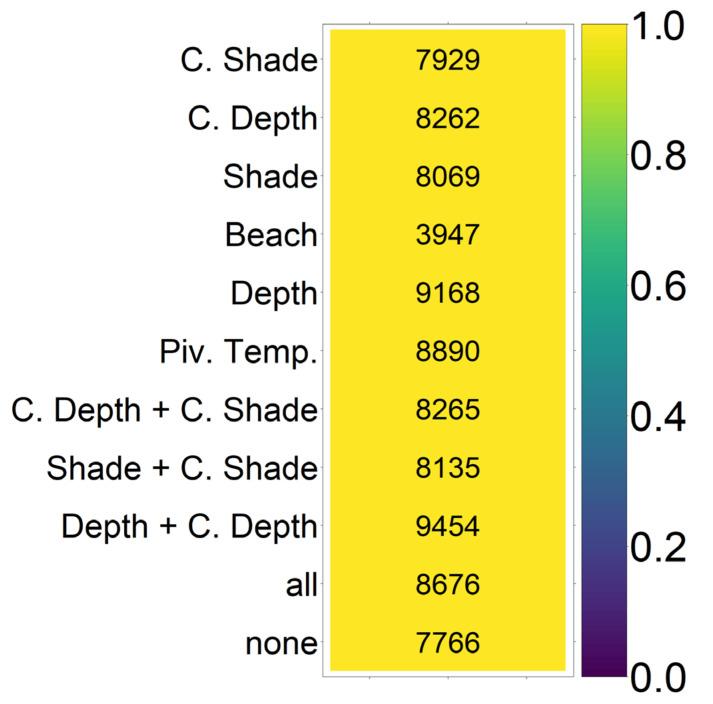
Results from the generation of a population for the southern Great Barrier Reef. Adult males were used as a source of migration to the nGBR for corresponding simulations. Colours indicate the proportion of replicates that survived for 1000 years. Numbers are the number of males retrieved for each setup.

The probability of meeting a male from the sGBR is, as a simplifying assumption, constant over time. Due to a lack of information about the extent of crossbreeding between populations, we simulated a range of different meeting probabilities. If a female encounters males from both populations, the nGBR male will fertilize all eggs. For the model, we created a sGBR population under the same conditions as the nGBR population, but always with a constant temperature of 29.61336 °C that would result in their current sex ratio of 0.67 [21] in the absence of evolution or conservation measures. Because the sGBR’s setup matched the one of the nGBR population they migrated to, they were potentially able to reach a 50:50 sex ratio again by evolving accordingly. This population was simulated for 1000 years to allow it to equilibrate. We collected all adult males from 100 simulations per setup in order to have a large pool of males available to migrate to the nGBR populations. Not all populations survived or had an equal proportion of males, and thus the total number of males to choose from differs between setups (Figure A6).

Note that we tested the effectiveness of each evolutionary mechanism individually at first, without the influence of conservation and/or migration. 

## 3. Results

### 3.1. Constant Temperatures

**Figure 3:**
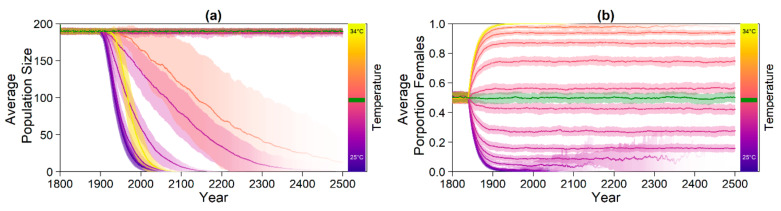
Model results without evolution of any trait and at various constant nest temperatures (different colours). (**a**) Average population size; (**b**) average proportion of females. Colour intensity indicates the number of populations that did not go extinct. Although extinct populations were included as zeros in population size averages, they were excluded from the calculation of the average proportion of females. The green line indicates replicates run at the pivotal temperature of 29.3 °C. Shaded areas show the standard deviation among replicates.

Keeping the temperatures constant over the timespan of the simulation and allowing no evolution led to at least 95% females for temperatures above 30.6 °C. Figure 3 shows average population size (a) and average sex ratio (b) over time. For both high and low temperatures, population size decreased over time, whereas it stayed roughly constant at temperatures around the pivotal temperature. Sex ratios were strongly dependent on temperatures.

**Figure 4:**
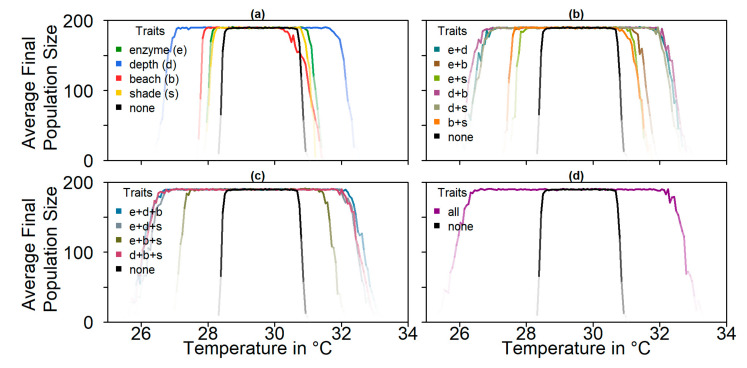
Average population size over 100 replicates at the end of the simulation (year 2500) depending on nest temperature, comparing all mechanisms and any combination of them. The black lines represent average population sizes without any evolution; coloured lines represent different evolutionary mechanisms. Colour intensity indicates the number of populations that did not go extinct: (**a**) one trait; (**b**) combination of two traits; (**c**) combination of three traits; (**d**) combination of all four traits.

We simulated populations with all four possible evolutionary mechanisms: nest depth, pivotal temperature, level of shade, and beach orientation, as well as their combinations (Figure 4). All mechanisms except beach orientation increased average population sizes at the end of the simulation for both colder and warmer temperatures. The most effective setup for survival at high temperatures was the combination of all evolutionary mechanisms. Among the single mechanisms, nest depth promoted population survival the most. Beach orientation was the least effective evolutionary mechanism for adapting to warmer temperatures.

### 3.2. Climate Change

**Figure 5:**
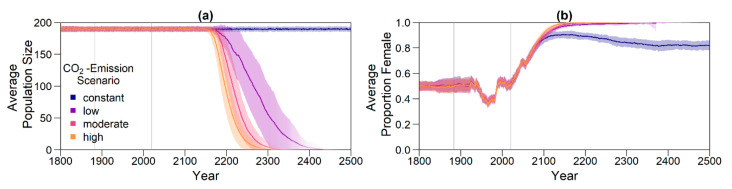
Results for the climate change simulation without evolvable traits. Intensity of the line represents proportion of surviving populations. Shaded areas indicate the standard deviation between replicates. Grey lines mark the points in time from which weather data (1883) and temperature predictions (2020) were used. Note that the effect of the change in temperature is visible in the sex ratio with a 40 year delay, as only adult individuals were taken into account for averaging. (**a**) Average adult population size over time; (**b**) proportion of females among adults over time. Each line in (b) represents the average of all non-extinct replicates at the respective time point.

For the climate-change simulation, there were four different scenarios (see Figure 1). Predictions for average population sizes and sex ratios with no adaptation to climate change are shown in Figure 5.

**Figure 6:**
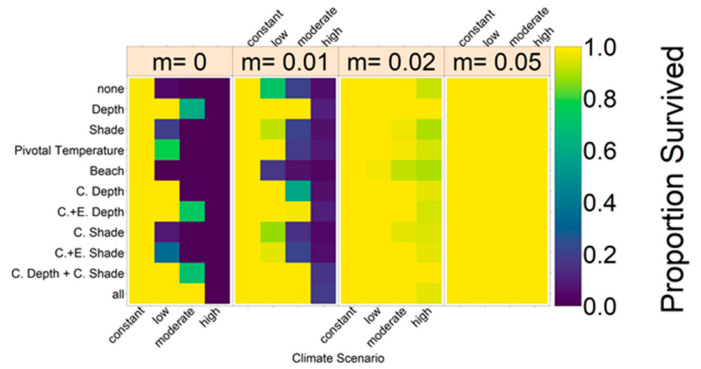
Overview of the proportion of populations in the various setups that did not go extinct by 2500. The left block represents the results without migration and the blocks to the right the results for different migration rates, *m* (indicated in top box, see Section 3.2.3). Colours indicate the proportion of populations that still persist in 2500. Each row depicts one evolutionary mechanism (C = conservation, E = evolution). Each column shows the result for one climate scenario. Sections show results for different migration rates between 0 and 0.05. Proportion of surviving populations are indicated by colours.

Without evolution, population size dropped dramatically within the next centuries for the moderate and high emission scenario, with the fastest decline for the high emission scenario (Figure 5a). For the low, moderate, and high scenario, almost all populations went extinct (Figure 6). With a constant level of CO_2_ emissions, the population size stayed stable close to carrying capacity. Figure 5b illustrates the sex ratio of the population over the years. Starting in the year 1883, when weather recordings were incorporated, sex ratio began fluctuating and eventually became more female-skewed when juveniles born during ca. 1990 to 2018 reached maturity 40 years later. Although for the constant emission scenario, the average final sex ratio was 0.82, the low, moderate, and high scenarios each reached a proportion of females of around 1. 

**Figure A7:**
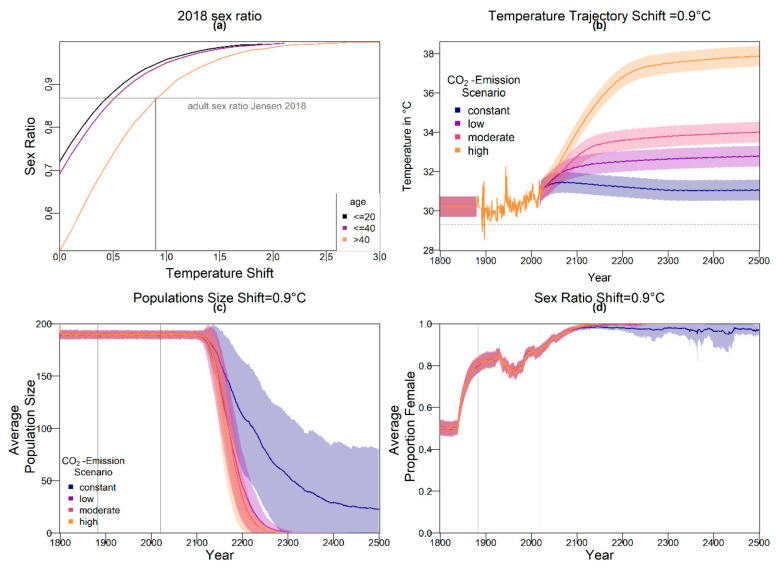
Choosing the shift in nest temperature relative to air temperature to generate the sex ratio found in Jensen et al. (2018) [21]. Panel (**a**) shows sex ratio in 2018 for adults (>40 years), and juveniles (≤40 years and ≤20 years), plotted against the additional increase in nest temperature added to the temperature used throughout the main text. Note that the juvenile classes overlap. We included them both because turtles at age 20 were categorized as adult-sized by Jensen et al. (2018), while we defined 40 years as maturing age [21]. In panel (**b**) we show the corresponding temperature pattern that resulted in an adult sex ratio closest to empirical observations [21]. Nest temperatures needed to be increased by 0.9 °C compared to our default scenario in order to generate 86.5% (86.8% observed [21]) females among adults (>40), 93.8% among individuals of up to 40 years of age, and 94.7% in those younger than 21 years. Trajectories show yearly average temperatures in parallel to Figure 1, and shaded areas are the standard deviation which was used to draw temperatures for individual replicates. Note that almost all nest temperatures exceed the pivotal temperature. Panels (**c**) and (**d**) show the resulting population size and sex ratio, with shaded areas being the standard deviation among replicates, parallel to Figure 5. Note how sex ratio increased early in the simulation, which appeared to be as soon as the starting population was replaced by individuals generated through the simulation.

Note that the reported sex ratio of adult nGBR turtles in 2018 was 0.86 female [21], whereas in the model it was closer to 0.59 at that time. This might have in part been due to the fact that we defined “adult turtles” as turtles that are 40 years and older, whereas Jensen et al. grouped turtles on the basis of size, and thus they counted them as adults around the age of 25 [21]. Adult turtles in 2018 in our model therefore had their sex determined before 1978, when the temperatures were not yet as high [21]. Because there was considerable uncertainty in the difference between air temperature and nest temperature, we introduced an additional temperature shift to try to match the sex ratio observed by Jensen et al. [21]. Results can be seen in Figure A11. The temperature shift corresponding to a sex ratio close to 0.86 led to a scenario where nest temperatures were almost never below and much more often well above the pivotal temperature. This would imply that the nGBR population was always heavily female-biased, even before any climate warming. This would mean that the population either escapes Fisher’s theories [29], or that the population has always been sustained by males from other populations. The results for this scenario predict that the population is on its way to extinction under all climate change scenarios, even the constant one (Figure A7).

#### 3.2.1. Evolution of Each Trait

**Figure 7:**
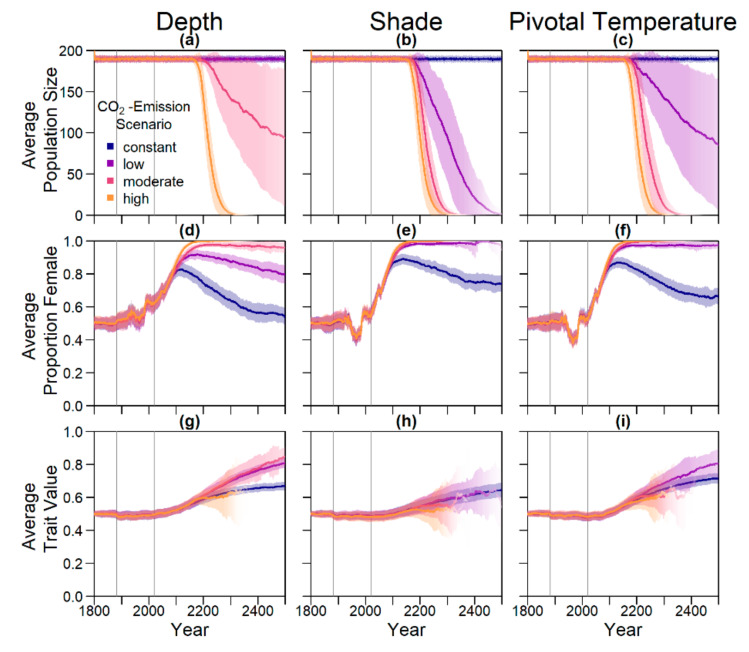
Results for evolution of nest depth, level of shade, and pivotal temperature. Intensity of the line represents proportion of surviving populations. Shaded areas indicate the standard deviation between replicates. Grey lines mark the points in time from which weather data (1883) and temperature predictions (2020) were used. Note that many effects are visible with a 40 year delay, as only adult individuals were taken into account for averaging. (**a**–**c**) Average population size over time; (**d**–**f**) average proportion of females among adults over time; (**g**–**i**) average genetic traits for nest depth, level of shade eggs are laid in, and pivotal temperature shift over time.

Figure 7 shows the results for the evolution of nest depth, level of shade, and pivotal temperature. Evolution of nest depth is the most effective mechanism, keeping the population stable close to carrying capacity for the constant and low emission scenario, whereas all populations in the high scenario and 21% in the moderate scenario still went extinct by 2500. Evolution of level of shade and pivotal temperature in the moderate and high climate change scenarios merely slowed down extinction compared to the case without evolution (Figure 5 and Figure 6). In the low emission scenario, evolution of pivotal temperature increased population survival from 2% to 77%, whereas it reached 20% when the level of shade was an evolvable trait. The sex ratios fluctuated according to the recorded temperatures in a similar way for all climate scenarios before changing according to the climate scenario and the respective evolutionary mechanism. After an initial rise in sex ratio starting in 2020, the increase in females can be reversed by evolution of traits (lower row). Average nest depth, level of shade, and pivotal temperature all increased for the three warmer emission scenarios. For the warmest scenarios, a sex ratio of 1 was reached before evolution of the respective trait could counteract the feminization.

**Figure 8:**
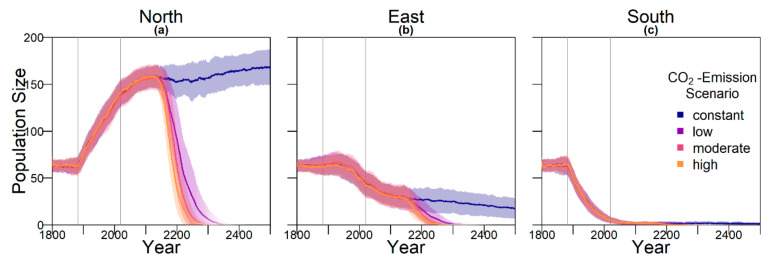
Population size on each of the nesting beaches. Intensity of the line represents proportion of surviving populations. Shaded areas indicate the standard deviation between replicates. Grey lines mark the points in time from which weather data (1883) and temperature predictions (2020) were used. Note that the effect of a change in temperature is only visible in the sex ratio with a 40 year delay, as only adult individuals were taken into account for averaging: (**a**) average population size on the northern beach, which was 0.7 °C warmer than the eastern beach; (**b**) average population size on the eastern beach, which was assumed to correspond to the respective baseline nest temperature; (**c**) average population size on the southern beach, which was 0.9 °C colder than the eastern beach.

The evolution of the beach orientation led to extinction in all replicates for the three climate warming climate scenarios (Figure 6 and Figure 8). Population size dropped on the eastern and southern beaches, whereas it increased on the warmest, northern beach, before the population went extinct. This was in contrast to the other evolutionary scenarios where long-term persistence appeared enhanced at least in the low emission scenario. Populations with evolving beach orientation fared even worse than populations without any evolving traits.

As under constant temperature, we also explored combinations of evolutionary mechanisms under climate change. An overview of population persistence in these can be found in Figure A6. Generally, the more evolutionary mechanisms that were combined, the higher the proportion of survival. Again, there was one exception—the evolution of the beach orientation led to a slightly higher extinction rate among populations.

**Figure A8:**
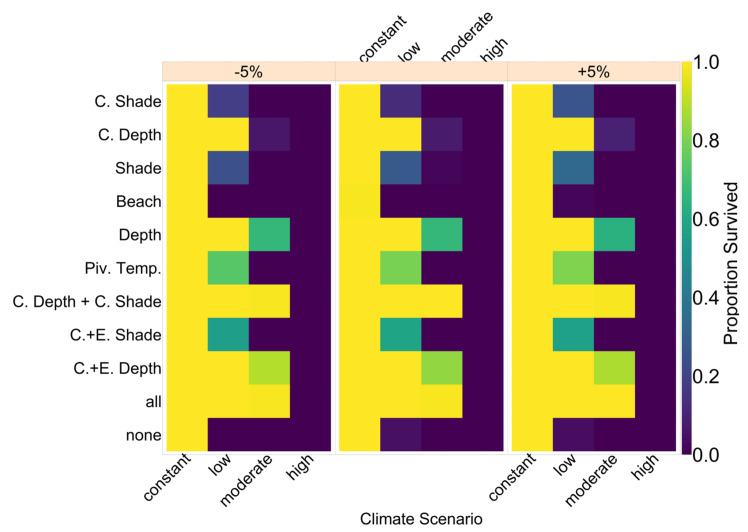
Robustness analysis for age at maturity. Results for varying parameters by ±5%. The *y*-axis shows each mechanism and any combinations thereof; the *x*-axis shows the parameters for each of the four climate scenarios. Colours indicate the proportion of replicates in which the population survived up until year 2500.

**Figure A9:**
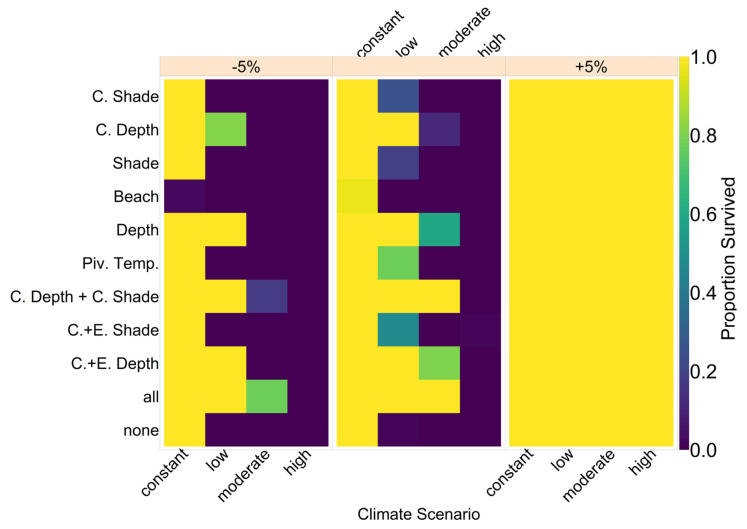
Robustness analysis for both juvenile and adult survival rates. Results for varying both parameters jointly by ±5%. The *y*-axis shows each mechanism and any combinations thereof; the *x*-axis shows the parameters for each of the four climate scenarios. Colours indicate the proportion of replicates in which the population survived up until year 2500.

**Figure A10:**
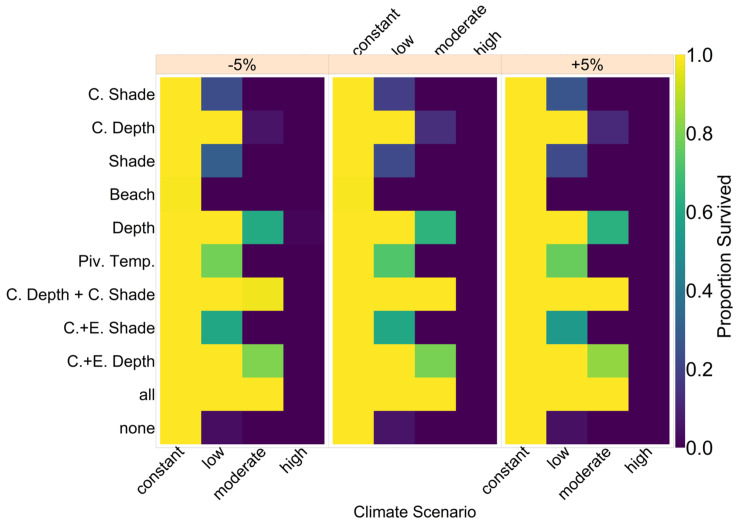
Robustness analysis for standard deviation of the Beta distribution representing mutation. Results for varying parameters by ±5%. The *y*-axis shows each mechanism and any combinations thereof; the *x*-axis shows the parameters for each of the four climate scenarios. Colours indicate the proportion of replicates in which the population survived up until year 2500.

**Figure A11:**
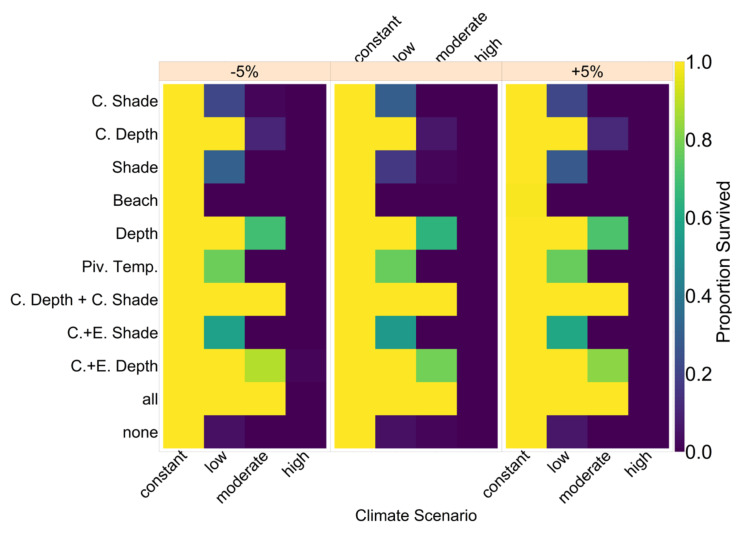
Robustness analysis for pre-industrial baseline nest temperature. Results for varying parameters by ±5%. The *y*-axis shows each mechanism and any combinations thereof; the *x*-axis shows the parameters for each of the four climate scenarios. Colours indicate the proportion of replicates in which the population survived up until year 2500.

In Appendix A, we explore the robustness of our results to changes in various parameters. The effect of different numbers of mating areas is shown in Figure A3. Population survival prospects decreased with an increasing number of mating areas, regardless of the evolutionary mechanism at hand. For fewer mating areas, population survival depended greatly on the chosen evolutionary mechanism. Additionally, we explored the sensitivity of our results to the following parameters: age at maturity (Figure A8), survival rate (Figure A9), mutation rate (Figure A10), and average pre-industrial (1800–1883) nest temperature (Figure A11). All parameters were varied by ±5%. The model was most sensitive to changes in survival rates (Figure A9). When increasing survival rate of both juveniles and adults by 5%, proportion of populations surviving to 2500 reached 100% for all possible scenarios and mechanisms, including the high emission scenario. This was because the yearly probability for an individual to die was then only 0.439%, making the average adult lifespan 227.79 years compared to 19.3 years (age of 59.3) in our default setup. Consequently, only three generations passed over the course of the simulation. Lowering survival rate by 5% did not have the same extreme effect, but population survival did decrease for all scenarios. For all other parameters, varying them did not lead to any unexpected or large differences in population survival. Moreover, we altered juvenile survival, that is, the survival probability from egg to mature adult (Figure A2). The model was most sensitive to changes in juvenile survival between 0.001 and 0.02. Increasing survival led to an increase in overall population survival probability for most emission scenarios. No juvenile survival rate that we tested enabled the population to survive the high emission scenario.

#### 3.2.2. Conservation Efforts

**Figure 9:**
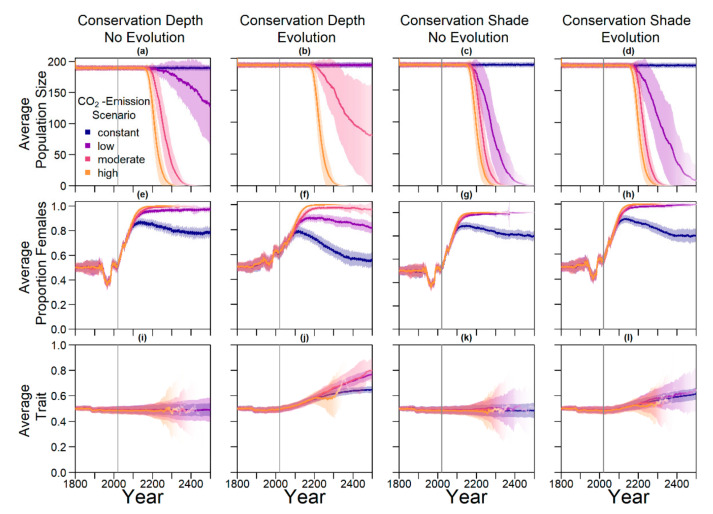
Results for conservation efforts combined with evolution of level of shade and nest depth. Grey lines indicate the start of conservation measures in 2020. (**a**) Average population size over time where every second nest was moved to 90 cm every 10 years, but nest depth was not an evolvable trait; (**b**) average population size over time where every second nest was moved to 90 cm every 10 years, and nest depth was an evolvable trait; (**c**) average population size over time where every second nest was moved to 30% shade every 10 years, but preferred level of shade was not an evolvable trait; (**d**) average population size over time where every second nest was moved to 30% shade every 10 years, but preferred level of shade was an evolvable trait; (**e**–**f**) corresponding average proportions of females among adults over time; (**i**–**l**) corresponding average trait values over time.

Figure 9 shows the results of conservation efforts, without and in combination with trait evolution. Conservation efforts were carried out every 10 years and were applied to every second nest (for other parameter combinations see Figure A5). Nests were set at a depth of 0.9 m and at a 30% level of shade. 

In the two left columns of Figure 9, we present the population size and sex ratio over time for conservation by manipulating nest depth with and without evolution. For the moderate, high, and low emission scenario, population size declined over time without evolution, whereas for the constant scenario it stayed at capacity. The higher the emission scenario, the quicker the decline. For the high and moderate emission scenario, population size dropped to zero before the year 2400. When combining conservation and evolution, average population size stayed close to carrying capacity for the constant and low scenario. Nest depth conservation alone increased the proportion of populations that survived until 2500 from 2% to 99% in the low and from 0% to 2% in the moderate emission scenario (Figure 6). When this trait additionally was evolvable, population survival increased to 100% for the low scenario and 74% in the moderate emission scenario. This pattern repeated in a less pronounced way for shade conservation in the low emission scenario, where 98% of populations went extinct without evolution or conservation, but with conservation efforts alone the proportion of surviving populations reached 9%, and combined with evolution, it reached 40%. Only for the high emission scenario did the combination of conservation and evolution not lead to any populations surviving to 2500.

Accordingly, sex ratio was less female-skewed when combining conservation and evolution. The average trait value stayed at 0.5 on average for nest depth and level of shade when there was no selection on traits, as expected. When combining conservation and evolution, the trait evolved towards similar levels as that without conservation (Figure 7).

Results for different rates of conservation measures are shown in Figure A5. The more nests the measures were applied to, the higher the proportion of surviving populations. Additionally, the more often an effort was carried out, the more effective it was. The most effective combination that we tested was carrying the measures out every two years and applying them to every second nest. We also simulated combinations of both conservation measures at the same time. Generally, when combining the evolution and conservation of nest depth and level of shade, the population survived all climate scenarios except the high one, no matter the degree of conservation. Even when only applying measures every 20 years to every 20th nest, a slight effect on population survival was visible. The high emission scenario, however, always had a survival probability of 0 (see Figure A5).

#### 3.2.3. Migration

For this part of the model, we created a southern GBR population that was used as a mating pool for the nGBR females. When creating these mating pools, not every scenario led to the same number of males in the southern GBR population (Figure A6). The main cause of reduction in population size was the choice of nesting beach, the same as for the northern GBR population. However, for every scenario there was always a sufficient number of males to serve as immigrants to the nGBR population.

We tested different levels of migration between the northern and southern Great Barrier Reef populations. The migration parameter rate *m* is defined as the probability for a northern female to meet a southern male in a given year. A higher migration rate led to an increased chance of population persistence for all evolutionary mechanisms and climate scenarios (Figure 6). Even small migration rates drastically improved the proportion of populations that survived until 2500 under all climate scenarios and for all schemes. For a migration rate of 0.05 or higher, 100% of populations survived in all setups. 

## 4. Discussion

The predictions for the four future climate scenarios without any evolution showed daunting results. The three higher CO_2_ emission scenarios led to extinction of the nGBR green sea turtle population. Currently, air temperatures have already increased by 0.8 °C compared to pre-industrial temperatures [18]. According to our model predictions, a further increase by 0.5 °C, elevating baseline nest temperatures to 30.6 °C, poses a threat to population survival (see Figure 4). However, our results suggest that evolution or conservation efforts might enable green sea turtles to recover from their current strongly skewed sex ratio, at least under the less extreme CO_2_ emission scenarios. 

On the basis of our assumptions, evolution of nest depth appeared to be the most promising route to evolutionary rescue. A major reason might be that it allows the largest reduction of nest temperature in our model (up to 2.8 °C), compared to the other traits that were assessed. With nest depth evolution, simulated populations survived until 2500 in the constant, low, and in most cases also the moderate CO_2_ emission scenarios. Evolution of the trait will counteract the shift in sex ratio towards females. For the high emission scenario, the temperature rose too quickly for evolution to keep pace. The sex ratio then reached 100% female and the population went extinct before a shift in nest depth could produce more males. Compared to nest depth, evolution of pivotal temperature and nest shading had similar yet weaker beneficial effects.

Contrary to our hypothesis that the option to deposit eggs at colder beaches would enhance population persistence, the maternal effect of nest site philopatry made the turtles go extinct faster than without any evolving traits. Northern beaches are closer to the equator and therefore the warmest, leading to more females being born there. Females from northern beaches will mate with males from southern beaches, but offspring will lay their eggs on the northern beaches as well. It is a vicious circle—more turtles on northern beaches leads to more females, leading to more eggs on northern beaches, leading to more females, and thus reducing male density even faster. Such a runaway process has already been suggested by Bull [54]. Via a similar effect, maternally inherited nest-site choice hindered adaptation to climate change in a mathematical model for painted turtle (*Chrysemys picta*) populations [55]. Indeed, it has been proposed that maternally inherited nest site choice can be one of the factors driving the evolution of environmental sex determination [56]. 

Although the level of philopatry in green sea turtles is generally considered extremely high (93–97% for the Sarawak population), some individuals do not return to their native beach [57]. For the model, increasing “mutations” of the nesting beach trait increased the proportion of surviving populations (see Figure A4). With a higher level of error, the possibility for having a few nests in colder temperatures and thus more males increases.

On a larger scale, there may be a similar effect between northern and southern GBR populations. Currently, there is no consensus on how frequent mating between the two populations is, but data suggest it might be quite common [24]. Because the southern population has a higher male ratio than the northern population, this could cause an increase in northern population size. A meeting frequency above 0.05 would, according to our model, largely increase the chance of population survival of the nGBR population in all emission scenarios. In loggerhead sea turtles in North America, there seems to be a similar relationship between northern and southern populations. Although the southern populations are highly female skewed there, the northern populations could potentially provide them with males [26].

**Figure A12:**
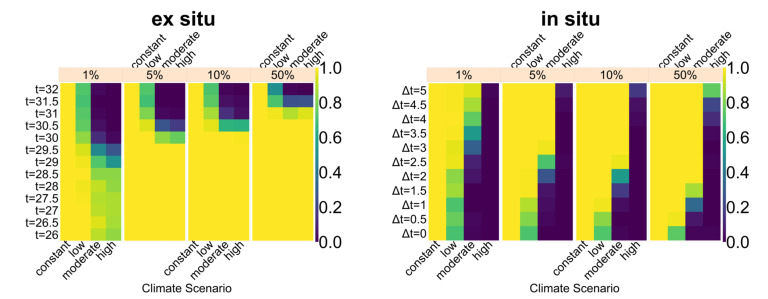
Effects of non-specific conservation measures. Colours indicate the proportion of simulated populations that survived to the year 2500. The orange boxes indicate what proportion of eggs was treated per year. Conservation was carried out starting in 2020 and applied every year. No additional evolution of traits, nest site philopatry, conservation measures, or migration was taken into account. On the left, eggs were incubated at a fixed and fully controlled temperature *t*. In order to achieve this, eggs would likely have to be removed from the nest and hatched in an incubator, which is why we labelled it ”ex situ”. On the right, nest temperatures were reduced by *Δt*, relative to the non-treated nests. The environmental temperature therefore still influences nest temperatures. Methods to do this without extracting eggs from their nests could be used, making us label this “in situ”. Sprinkling the sand with (cold) water could be one way to achieve this.

Our model results also show some interesting possibilities for conservation efforts. Putting the nests deeper into the ground by hand every few years might shift the sex ratio enough to ensure a ratio of males that is high enough to sustain the population. Shading nests may, however, be easier to implement. Unfortunately, in the model, it was not as effective as altering the nest depth. In the data used for nest temperatures under different levels of shade, the maximum amount of shade possible was at 30% [35]. Possibly more shade could be provided to further lower the temperature. However, implementing sun protection may prevent rainfall from reaching the nest. This will then make the nest warmer than its surroundings instead of colder [35], and thus any conservation must be implemented carefully and underlie constant supervision to ensure temperatures are actually lowered. In Figure A12, we aim to give an idea of how much cooling of nests by in situ or ex situ methods would be necessary in order to improve the chances of population survival. By in situ we mean a situation where nests would still be exposed to the outside temperatures, but nest temperatures could be reduced by a method of choice (for example sprinkling water on the nests). With the ex situ methods, we assume that the effect of outside temperature is eliminated completely, for example by extracting nests and putting them in incubators with controlled temperatures around 29.3 °C until hatching. Results indicated that keeping 5% of all nests at a temperature of at most 29.5 °C every year ex situ would lead to a survival probability of 100% for all climate scenarios. With in situ measures, lowering nest temperatures by up to 5 °C did not enable population survival for the high emission scenario. Reducing nest temperatures by 3 °C for 5% of all nests, however, did ensure survival for the constant, low, and moderate emission scenarios.

The model suggests that artificially changing nest depth or shade level does not interfere much with the natural evolution of these traits, as nest depth still increased with increasing temperatures, for instance. However, the average nest depth trait value at the end of the simulation decreased by 3.8% when in combination with conservation compared to evolution only. This suggests that anthropogenic conservation efforts reduce selection pressure on the trait, but only to a very small degree, which may also be due to stochastic effects. For level of shade, the trait even increased by 0.15% relative to the scenario without evolution, which was likely due to noise.

Although the evolution of nest depth showed promising results, at least for the low and potentially the moderate CO_2_ emission scenarios, there was one big caveat—we do not know whether nest depth is a hereditary trait at all, as there are currently no data available on variation in nest depth between individual turtles in a population. One study suggests that nest depth may be correlated with limb size, and thus larger turtles generally dig deeper nests [58]. Therefore, if body size or growth rate is a heritable trait, then nest depth might be as well. However, even if nest depth is a hereditary trait, more factors play an important role in determining the temperature of the nest besides the depth. The grain size of the sand on a nesting beach as well as its colour, the moisture of the ground, and levels of rainfall and shade all influence the temperature of nests. Moreover, rising sea levels could lead to erosion of beaches, potentially limiting possible nest depth and nest position choice [59]. For example, in loggerhead sea turtles, high humidity seems to lead to higher male frequencies even at high temperatures [40]. Moreover, the temperature of the ground depends on the climate in the months before the mating season. Towards the end of the mating season, the ground is heated up by the summer [45]. As temperatures drop, the ground stays warmer, and thus this case, the deeper the nest is in the ground, the warmer it is. In addition to these factors, nest depth might be limited by the ability of hatchlings to reach the surface and the durability of eggs themselves.

For pivotal temperatures, data for other turtle species suggest that there is substantial heritable variation within populations [37]. One of the genes underlying pivotal temperature is the cold-inducible RNA-binding protein (CIRBP) [60]. CIRBP has two alleles, one of which is thermosensitive whereas the other is not [38]. The expression pattern of CIRBP within developing gonads shows that it is able to mediate temperature effects on the bipotential gonads, effectively shifting the pivotal temperature [60]. Allele frequencies differ between snapping turtle populations and are correlated with pivotal temperatures [60]. This suggests potential for adaption. However, to our knowledge, there are currently no data on CIRBP allele frequencies in *Chelonia mydas*. The way the algorithm is set up in the model allows for the population to shift its pivotal temperature by up to 1 °C.. However, it is unclear at what spatial scale pivotal temperature could adapt. In green sea turtles at Ascension Island, there was no evidence for local adaptation in pivotal temperature across beaches with different sand temperature [61].

For the other evolving traits in our model, not much is known about the genetic basis and heritability. To improve the accuracy of the model, it is necessary to establish more data on these traits. Field studies on green sea turtles unfortunately take a long time to produce data [17].

In addition to the traits considered here, green sea turtles could potentially shift their breeding season in response to climate warming. In some bird species, a warmer climate has led to an earlier onset of breeding seasons [62]. For other turtle species, variation in nesting phenology has been documented across space and time [2,63,64]. During the end of spring, the ground is not as heated as during summer [45]. Thus, a shift in breeding season might mitigate the effects of climate change on the sex ratio of the turtles. 

In both painted turtles, *Chrysemys picta*, and loggerhead sea turtles, *Caretta caretta*, females showed plasticity in the date of first nesting depending on year-to-year climatic variability. Warmer winters lead to an earlier onset of nesting. A possible mechanistic explanation for a change in nesting date might be that a warmer winter allows *C. picta* to emerge earlier from hibernation, or provide them with more basking opportunities and thus influence the rate of egg development [65]. Another study on *C. picta* suggests that the level of shade provided for a nest might be a behavioural plastic trait [58].

All of these studies suggest that behavioural plasticity might be a possible way for species to alleviate the effects of rising temperatures. However, green sea turtle nesting dates do not seem to respond as much to year-to-year variation in sea surface temperatures [43]. This might be because of their different diet or because they only seldomly display hibernating [66] or basking behaviour [67]. Furthermore, the fact that nGBR green sea turtle sex ratios have already become heavily female-biased [21] suggests that green sea turtles have limited phenotypic plasticity for nest-site choice or nesting date in response to temperature.

Our results were based on historical data and predictions for average December–March temperatures and we took into account interannual variation in temperatures, but not variation in temperatures within breeding seasons. Future modelling efforts could also include finer-scale temporal variation and small-scale spatial variation in temperatures. If at least some nests experience cold enough temperatures during the critical developmental time window, the resulting males could have a strong effect on the population’s persistence time. On the other hand, temperature fluctuations have also been suggested to have a feminizing effect, at least in painted turtles and red-eared slider turtles [37,68].

Another aspect that might potentially influence population survival but is not included in the model are the effects of inbreeding and small population size. When males become scarce and the same few males mate with most females, offspring are going to be more related than usual. This could lead to inbreeding depression and loss of genetic variation and evolutionary potential. Additionally, there are currently no data available as to how many females a male is able to mate with in nature and how the mate finding process works and depends on the density of males and females. Filling these gaps in knowledge could greatly improve the precision of model predictions in the future. 

Although we focused on the effect of rising temperatures on primary sex ratios within the population, there are a number of other factors that make marine turtles vulnerable to climate change. Depending on the age of a turtle, they may be affected by alteration of rainfall, storms and cyclones, rising sea levels, alteration of winds and ocean currents, alteration of large-scale ocean-atmosphere patterns, and ocean acidification [69].

Several previous studies have attempted to make predictions for the fate of green sea turtles and other species with temperature-dependent sex determination under climate change. For a West African green sea turtle population, Patrício et al. predicted that males will be produced until 2100, even in the most extreme climate change scenarios [70]. The main difference compared to our scenario appears to be that sand temperatures in this location are generally cooler, especially in forested areas, such that the current sex ratio among hatchlings there is currently at only 52% female [70]. A study by Fuentes and Porter [71] compared two different modelling approaches to predicting soil temperatures in green sea turtle nesting grounds at the nGBR—a correlative model and a more complex and mechanistic microclimate model that takes into account climate maximum and minimum data (e.g., air temperature, wind speed, humidity, percentage of cloud cover) for arbitrary time intervals, such as monthly, weekly, or daily, and physical properties of the soil (e.g., thermal conductivity, density, specific heat, and substrate reflectivity). In the correlative model, the authors found that using both air and sea surface temperatures to predict soil temperature led to a more accurate prediction of soil temperature than using either air or sea surface temperatures on their own [34]. They found that microclimate and correlative modelling approaches led to different soil temperatures and therefore different sex ratio predictions for the nGBR population, but agreed in that a complete feminization is inevitable should temperatures keep rising as predicted by most emission scenarios by the IPCC [18]. However, they assumed that the sex determination system remains constant over time and does not evolve. A productive direction for future work could be to include variables such as sea surface temperature, rainfall, cloud cover, slope, aspect, reflectivity, wind speed, and humidity in an eco-evolutionary model such as ours in order to make more accurate predictions for the fate of green sea turtle populations.

Another species with temperature-dependent sex determination that is at risk because of climate change is the tuatara (*Sphenodon punctatus*), a native New Zealand reptile that has also a relatively long generation time. Unlike green sea turtles, tuatara currently have small current population sizes and low levels of genetic variation. Tuatara have the opposite pattern of TSD, with female hatchlings at cold temperatures and male hatchlings at warm temperatures. A biophysical microclimate model predicts that under maximum warming forecasts for 2080, almost all nests will produce 100% male hatchlings [72]. A shortage of females has even more immediate demographic consequences than a shortage of males. This is because warmer temperatures lead to a rise in females. Initially, this rise in females causes a rise in population size, as few males can mate with many females [73]. However, when temperatures continue rising and males become scarce, matings become scarcer as well, eventually driving the population to extinction. On the other hand, for cool temperatures, an increase in males leads to a steep population decline. In a study comparing the extinction risk for species with environmental sex determination under climate change on the basis of a set of criteria, the tuatara received the highest risk score [2]. Because of their MF sex determination system and the higher population size and levels of genetic variation, green sea turtles, which were not included in that study, would likely receive a somewhat lower risk score. However, our results show that even if we optimistically assume substantial heritable variation in relevant traits, there are limits to ability of this species to adapt to a warming climate.

## 5. Conclusions

To conclude, modelling green sea turtle population ecology and evolution shows some possible ways to support green sea turtle survival. The turtles may be able to adapt to climate change if the CO_2_ emissions stay within a low to moderate range. However, model assumptions on heritability of traits and variance within the population remain to be empirically tested to ensure model accuracy. In the meantime, anthropogenic conservation measures may support population survival without compromising evolution.

## Supplementary Materials

The following are available online at https://www.mdpi.com/2073-4425/11/5/588/s1. We provided a zip folder containing simulation code: T3W.R, analysing script: SOLVER3W.R, figure creation script: ALL_FIGURES.R, temperature data for simulation: temperature.RData, summary of files: RFiles_readme.txt.

## Appendix A

Calculation of the TSD equation (Equations (A1) and (A2)) *a* is the upper limit, and *b* is the constant of proportionality. They were calculated using the following data by Limpus [17]—at 29.3 °C a 0.5 sex ratio of females is produced, so *t_piv_* = 29.3. For temperatures going towards infinity, the sex ratio of females will go towards 1, which requires *a* = 1. At 28 °C the sex ratio is 0.05 female [17], that is,

(A1) $$0.05=\frac{1}{1+e^{\frac{-\left(28-29.3\right)}{b}}},$$

leading to

(A2) $$b=\frac{1.3}{\ln\left(19\right)}=0.4424779,$$
